# Arginine and Arginine-Rich Peptides as Modulators of Protein Aggregation and Cytotoxicity Associated With Alzheimer’s Disease

**DOI:** 10.3389/fnmol.2021.759729

**Published:** 2021-10-28

**Authors:** Somayra S. A. Mamsa, Bruno P. Meloni

**Affiliations:** ^1^School of Molecular Sciences, Faculty of Science, The University of Western Australia, Perth, WA, Australia; ^2^Perron Institute for Neurological and Translational Science, QEII Medical Centre, Perth, WA, Australia; ^3^Centre for Neuromuscular and Neurological Disorders, The University of Western Australia, Crawley, WA, Australia; ^4^Department of Neurology, Sir Charles Gairdner Hospital, QEII Medical Centre, Perth, WA, Australia

**Keywords:** arginine, aggregation, Alzheimer’s disease, peptides, amyloid-beta (Aβ), tau & phospho-tau protein

## Abstract

A substantial body of evidence indicates cationic, arginine-rich peptides (CARPs) are effective therapeutic compounds for a range of neurodegenerative pathologies, with beneficial effects including the reduction of excitotoxic cell death and mitochondrial dysfunction. CARPs, therefore, represent an emergent class of promising neurotherapeutics with multimodal mechanisms of action. Arginine itself is a known chaotrope, able to prevent misfolding and aggregation of proteins. The putative role of proteopathies in chronic neurodegenerative diseases such as Alzheimer’s disease (AD) warrants investigation into whether CARPs could also prevent the aggregation and cytotoxicity of amyloidogenic proteins, particularly amyloid-beta and tau. While monomeric arginine is well-established as an inhibitor of protein aggregation in solution, no studies have comprehensively discussed the anti-aggregatory properties of arginine and CARPs on proteins associated with neurodegenerative disease. Here, we review the structural, physicochemical, and self-associative properties of arginine and the guanidinium moiety, to explore the mechanisms underlying the modulation of protein aggregation by monomeric and multimeric arginine molecules. Arginine-rich peptide-based inhibitors of amyloid-beta and tau aggregation are discussed, as well as further modulatory roles which could reduce proteopathic cytotoxicity, in the context of therapeutic development for AD.

## Introduction

Alzheimer’s disease (AD) is a progressive, neurodegenerative disease estimated to affect over 44 million people worldwide, with a devastating impact on patients, their loved ones and caregivers, as well as vast social and economic consequences (Alzheimer’s Association, 2021). As the single biggest risk factor for AD is age, the prevalence of AD continues to increase as the average life expectancy rises; by 2050, it is predicted that over 100 million people worldwide will have the disease (GBD 2016 Dementia Collaborators, 2019). Clinically, AD is characterised by memory loss, impaired cognition, changes in mood, affect and behaviour, and a decline in the ability to carry out everyday tasks. These symptoms arise gradually, developing over the course of several years from mild cognitive impairment to dementia of increasing severity. Due to the progressive and insidious nature of AD, limited availability of tools for definitive diagnosis (Gofton and Weaver, 2006), and a lack of efficacious and disease-modifying therapeutics (Weller and Budson, 2018), AD remains a significant issue in global health.

AD is characterised by the development of proteopathies, specifically cytotoxic aggregates of amyloid-beta and tau. A range of therapeutic approaches for AD-associated proteopathies are therefore in various stages of development, including enzyme inhibitors targeting the production pathway of amyloid-beta (Kumar et al., 2018), gene silencing technologies to limit the expression of pro-aggregatory mutant tau proteins (Miller et al., 2004), kinase inhibitors aimed at preventing the pro-aggregatory hyperphosphorylation of tau, passive and active immunotherapies developed to drive protein clearance, and small molecule inhibitors of protein aggregation. These strategies are comprehensively reviewed in the literature (Hardy and De Strooper, 2017; Congdon and Sigurdsson, 2018; Pedrini et al., 2019). It remains unclear whether the proteopathies observed in AD are causative or consequential with regards to other aspects of AD pathogenesis, such as inflammation, neurovascular dysfunction, and metabolic disorders. Although the precise, mechanistic contributions of protein aggregation to the pathogenesis of AD are still contested, targeting protein aggregation remains a central priority in drug development; the recent approval of the amyloid-beta-lowering human monoclonal antibody Aducanumab by the United States Food and Drug Administration (FDA) represents the first drug to be approved by the FDA for the treatment of AD in 18 years.

Broadly, peptide-based therapeutics represent a particularly desirable class of candidates for pharmacotherapy, as peptides can be designed with high target specificity and bioactivity, and further optimised for increased safety, tolerability, efficacy and stability through sequence and structural modifications (Di, 2015; Räder et al., 2018; Evers et al., 2019). In the case of protein aggregation, the sequence and structural features of target proteins can be used to inform the rational design of peptides for binding and inhibition. Additionally, methods for screening peptide-based compounds are well-developed, including a variety of predictive *in silico* tools for assessing potential interactions between peptides and proteins (D’Annessa et al., 2020). As a general class, peptide-based inhibitors of protein aggregation developed for AD have been reviewed in detail by Goyal et al. (2017), Kumar and Sim (2014), and Funke and Willbold (2012). Despite their advantages, challenges in the development of anti-aggregatory peptides include lack of membrane permeability and difficulty in penetrating the blood-brain barrier. Cationic arginine-rich peptides (CARPs) demonstrate a particular ability to cross cellular membranes as well as the blood-brain barrier (Mitchell et al., 2000; Schmidt et al., 2010; Allolio et al., 2018), rendering them popular “carrier” molecules for a range of therapeutic “cargo” such as oligonucleotides, peptides and proteins (reviewed by Habault and Poyet, 2019). However, the aggregation-modulating properties of arginine itself may be favourable for peptide drugs targeting proteopathies. Here, we discuss the unique properties of arginine in modulating protein aggregation, as well as arginine-rich peptides, and peptides which have employed arginine as a key residue, in targeting the proteopathies associated with AD.

## Arginine

### Structure and Physicochemical Properties of the Arginine Monomer

**Figure 1:**
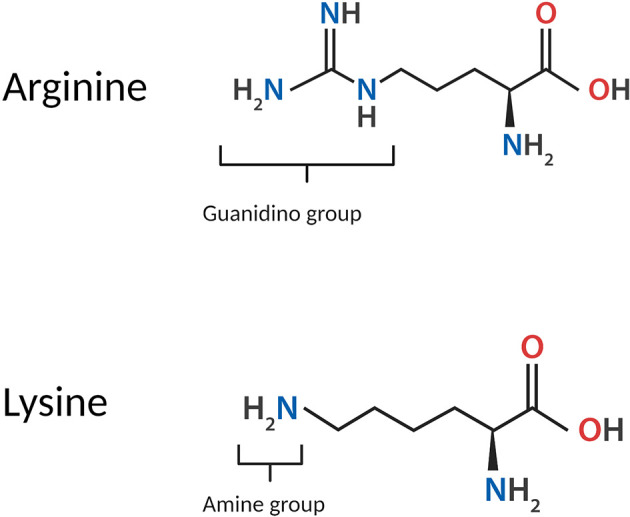
Structure of the arginine monomer in comparison to lysine.

Arginine is one of two basic amino acids, alongside lysine, which is consistently protonated at physiological pH. The structure of arginine is highly unique; arginine monomers are comprised of a polar, hydrophilic head group conjugated to a hydrophobic body, and an aliphatic side chain capped with a guanidino group. At physiological pH, the carboxyl moiety of arginine is deprotonated, while protonation of both the amino group into an amide and the guanidino group into the cationic guanidinium moiety, confers the overall cationicity of the molecule to a net charge of +1. In proteins, the guanidinium moiety contributes extensively to the intra- and inter-molecular associations of arginine residues by imparting a strong capacity for electrostatic interactions such as hydrogen bonding. The structure of arginine in comparison to lysine is shown in Figure 1.

Guanidinium itself is a planar molecule comprised of three amino groups conjugated to a central carbon atom; these three amino groups facilitate the formation of bidentate hydrogen bonds in three directions, enabling arginine to participate in a higher number of electrostatic interactions compared to lysine. Guanidinium also contributes to several distinctive properties of arginine and its behaviour in biological systems. The side chain of arginine is able to remain protonated under all physiological conditions and in even highly basic solutions (Xu et al., 2017), as the guanidinium moiety carries a highly stable, delocalised cationic charge with a pKa of 13.8 (Fitch et al., 2015) owing to resonance stabilisation. Due to its remarkable chemistry and emergent properties, guanidinium has been the subject of extensive study. The high stability of guanidinium is attributed to its Y-conjugated, quasi-aromatic structure with six delocalised pi electrons, which was regarded by Gund (1972) as a novel form of aromaticity. Additionally, the side chain of arginine is conferred partial hydrophobicity by the hydration structure of guanidinium; guanidinium is poorly hydrated above and below its plane, retaining only in-plane hydration (Mason et al., 2003, 2004).

### Emergent Properties, Self-association and Clustering

Notably, the guanidinium moiety of arginine imparts a strong tendency for self-association. While it is a basic tenet of physical chemistry that ions with like charges repel each other, guanidinium ions in solution are able to overcome the Coulomb repulsion typically driving this effect and form thermodynamically stable pairs (Vazdar et al., 2018). This behaviour also extends to arginine; Shukla and Trout (2010) observed the tendency of arginine monomers to form clusters in solution, through three dominant modes of self-association: guanidinium-to-guanidinium stacking; bonding of the guanidinium moiety of an arginine monomer to the carboxylate group of another; and bonding of the arginine C-terminus to the N-terminus of an adjacent arginine monomer. The ability of arginine to form stable clusters has largely been attributed to the hydrogen-bonding ability of guanidinium (Li et al., 2010).

Computational simulations have also demonstrated like-charge pairing of di-arginine peptides through the association of their side chains, while the NH^4+^-containing side chains of di-lysine peptides do not exhibit any attraction to each other (Vazdar et al., 2018), evidencing the critical role of the guanidinium moiety in arginine-arginine interactions. Longer, poly-arginine peptides are also attracted to each other in solution. The self-association of polyarginine-10 (R10) peptides was demonstrated by Tesei et al. (2017) through computational modelling and experimentally confirmed by small-angle X-ray scattering experiments. The guanidinium cations of arginine side chains throughout R10 were found to associate with the corresponding guanidinium groups on pairing R10 molecules to form like-charge ion pairs, while the guanidinium cation of the ninth residue was also found to bind the negatively charged C-terminus of the adjacent R10 peptide through the formation of an intermolecular salt bridge (Tesei et al., 2017; Vazdar et al., 2018). The latter mechanism functions as an “adhesive patch” between two R10 peptides, a binding motif absent in the interactions between two molecules of the similarly cationic peptide polylysine-10 (K10), which only repels itself in solution (Tesei et al., 2017; Vazdar et al., 2018).

The formation of like-charge guanidinium pairing between arginine residues is also likely to impart stability in the tertiary structure of proteins; a number of biologically occurring protein structures are known to contain arginine residues oriented in close proximity to each other in three-dimensional space, with these steric arrangements enabled by the formation of guanidinium pairs within the structure (Tesei et al., 2017; Vazdar et al., 2018).

At physiological pH, where arginine is protonated, self-association of arginine also facilitates the formation of dynamic arginine clusters bound to protein surfaces. *In silico*, arginine ions bind to the surface of proteins such as lysozyme through interactions mediated by their guanidinium and carboxyl groups; while bound, the remaining guanidinium or carboxyl group of each arginine monomer interacts in a head-to-tail orientation with the carboxyl or guanidinium group of another arginine molecule in solution, which itself is able to interact with another arginine molecule through the same mechanism (Vagenende et al., 2013). Arginine clusters remain bound to proteins for a remarkably long period of time, and are thus considered to “extend” the surface of the protein they are bound to (Vagenende et al., 2013); indeed, experimental results confirm that binding of arginine ions to a protein *in vitro* results in a size increase of the protein detectable by chromatography (Vagenende et al., 2013). Corollary to this, it is expected that clusters of arginine can alter the physicochemical properties of the protein they are bound to, including charge and hydrophobicity, and could therefore alter their properties *in vivo*, such as solubility, stability and activity.

### Effects of Arginine on Protein Aggregation

It is well-established that arginine modulates protein aggregation, acting as a molecular cosolvent and “chemical chaperone” in solution (Sharma et al., 2013). Arginine is commonly used as an additive to maintain protein stability in the biopharmaceutical industry and has conventionally been regarded as an aggregation suppressant (Golovanov et al., 2004; Arakawa et al., 2006; Ghosh et al., 2009). Shiraki et al. (2002) evaluated the effects of 15 amino acids on lysozyme under the conditions of thermal unfolding- and dilution-induced, aggregation; among them, arginine was found to be the most effective suppressor of aggregation, increasing protein solubility. Arginine has also proven particularly useful for solubilising membrane proteins otherwise prone to aggregation (Arakawa et al., 2011). Notably, the effects of arginine on proteins in solution are unique even in comparison to guanidine (Arakawa et al., 2007).

A number of experimental and computational studies have aimed to develop a mechanistic understanding of the molecular processes underlying the suppression of protein aggregation by arginine. It was previously reported by Das et al. (2007) that the self-association of arginine gives rise to hydrophobic surfaces through alignment of its methylene groups, which are then driven by the hydrophobic effect to bind the exposed hydrophobic residues of unfolded and intrinsically disordered proteins. Das et al. (2007) pertinently observed that the amyloid-beta peptide exhibits increased solubility and decreased fibril formation in the presence of arginine, and concluded, from their analyses, that these effects were due to hydrophobic surfaces of arginine clusters masking the pro-aggregatory hydrophobic residues of amyloid-beta monomers, preventing their self-association. However, while arginine does form hydrophobic columns along its crystallographic axis in crystal structures, molecular dynamics simulations subsequently performed by Shukla and Trout (2010) did not support the finding of methylene alignment in solution. While Shukla and Trout indeed confirmed the assembly of supramolecular arginine clusters, observing the formation of dimers and higher order n-mers at physiological pH, no hydrophobic surfaces were formed in these clusters through methylene alignment as proposed by Das et al. (2007).

Furthermore, Shukla and Trout (2010) also observed strong preferential interactions between arginine and the aromatic residues of their model proteins, mediated by cation-pi stacking; these observations were consistent with previous results by Arakawa et al. (2007), which demonstrated that while both arginine and guanidine displayed a strong affinity for most protein residues, their affinities were particularly strong for aromatic residues, which are generally hydrophobic. Tsumoto et al. (2004) had also previously observed that the aggregation-suppressive effects of arginine, which could not be explained through either surface tension effects or preferential interactions alone, were substantially mediated through the interactions of arginine guanidinium groups with the side chains of protein tryptophan residues. In the folded state of proteins, hydrophobic residues such as tryptophan are internalised or “buried” from the protein surface, while their externalisation in protein unfolding promotes aggregation; thus, arginine could indeed have a noticeable effect on the aggregation of unfolded or disordered proteins by binding hydrophobic surfaces and preventing their self-association, albeit through different molecular mechanisms than those proposed by Das et al. (2007).

Interestingly, however, Shukla and Trout (2010) reported that interactions between arginine and aromatic residues were insufficient to fully explain the effect of arginine on protein aggregation; in their study, the overall effect on aggregation was disproportionate when considering the relatively low number of aromatic residues comprising their model proteins. This led the authors to consider additional mechanisms, particularly the accumulation of arginine molecules on protein surfaces to form dynamic clusters; it was concluded that these clusters were able to “crowd out” the model proteins, preventing their self-association. Further research led to the classification of arginine as a “neutral crowder” in this context (Baynes et al., 2005). A comparative study by Schneider and Trout (2009) on the effects of arginine hydrochloride and guanidine hydrochloride on the aggregation of bovine serum albumin (BSA), lysozyme and α-chymotrypsinogen revealed that arginine had a unique effect: it was neither attracted to nor repelled from the protein surface. However, Schneider et al. (2011) later described a shift in the preferential interaction of arginine at high concentrations, from neutral to highly excluded, concluding that the “neutral crowder” effect was not able to completely describe the behaviour of salt forms of arginine such as arginine hydrochloride. Additionally, *in silico* analysis by Li et al. (2010) demonstrated the ability of arginine to solubilise hydrophobic and aromatic moieties by forming a “cage-like” solvation layer around the molecules. The mechanisms of aggregation suppression by arginine are therefore complex, involving not only interactions between arginine and proteins, but interactions between arginine and other arginine molecules; this tendency to form self-associative assemblies is an important factor in the aggregation-modulating effects of arginine.

Continued studies over the past decade, however, have demonstrated the effects of arginine on protein aggregation vary depending on its context and concentration, and are not always suppressive. Arginine potently inhibits the aggregation of lysozyme (Matsuoka et al., 2007; Ito et al., 2011), and porcine and mink growth hormones (Cirkovas and Sereikaite, 2011); monomeric arginine prevents the oligomerisation of insulin (Varughese and Newman, 2012; Březina et al., 2018; Haghighi-Poodeh et al., 2020), and di-arginine peptides exhibit even higher efficacy for suppressing insulin aggregation (Nuhu and Curtis, 2015). At physiological pH, arginine hydrochloride also suppresses the aggregation of immunoglobulin G1 (IgG1), with this effect attributed to the interactions between arginine and hydrophobic IgG1 residues (Fukuda et al., 2014). However, arginine and its derivatives arginine amide and arginine ethylester were found to alter the aggregation pathway of BSA, inducing the formation of larger BSA aggregates (Borzova et al., 2017) rather than inhibiting aggregation *per se*. Additionally, high concentrations of arginine suppress the aggregation of α-lactalbumin, while low concentrations are known to substantially alter its aggregation pathway, resulting in a distinctive aggregate morphology (Smirnova et al., 2013). These varied observations indicate the effects of arginine on protein aggregation are more complex and diverse than previously established. Thus, arginine is perhaps best described as a “complex molecular cosolvent”, and the molecular context of arginine greatly influences the manner in which it modulates the behaviour of proteins in solution.

## Proteopathies Associated with Alzheimer’s Disease

Until the 1990s, the dominant theory of AD focused on the “cholinergic hypothesis” which posited the impairment of cholinergic neurotransmission as the primary cause of cognitive decline observed in patients with AD (reviewed by Francis et al., 1999). However, subsequent bodies of evidence have strongly implicated amyloid-beta deposition as a central event in the development of AD, regarded as the “amyloid cascade hypothesis” (first proposed by Hardy and Higgins, 1992; reviewed by Barage and Sonawane, 2015, and more recently by Ricciarelli and Fedele, 2017). The minimal correlation between cerebral amyloid-beta load and the severity of cognitive decline observed in patients with AD (Nelson et al., 2012), however, has led to an increased focus on the role of tauopathy in the pathogenesis of AD. Moreover, the presence of cerebral amyloid-beta plaques is not always concomitant with impaired cognition (Arboleda-Velasquez et al., 2019). The development of amyloid-beta pathology is therefore considered necessary, yet insufficient, for the progression of AD. Broad evidence from laboratory, preclinical and clinical studies suggests that amyloid-beta aggregation can also drive the progression of tauopathy (reviewed by Stancu et al., 2014; Hanseeuw et al., 2019), which is increasingly believed to play a key role in cognitive decline (Nelson et al., 2012; Di et al., 2016; Digma et al., 2019). The precise, mechanistic roles of these proteopathies in the aetiology and pathogenesis of AD are the subject of extensive research and debate; however, pathogenic protein aggregation remains strongly implicated in the disease (Lovestone and McLoughlin, 2002; Thal and Fändrich, 2015; Jouanne et al., 2017; Gandhi et al., 2019; Johnson et al., 2019). We will briefly outline the characteristics of amyloid-beta and tau and their aggregation pathways.

### Amyloid-Beta

Amyloid-beta peptides are 38- to 43-residue peptides resulting from the sequential cleavage of amyloid precursor protein (APP) by the secretase family of enzymes (Crescenzi et al., 2002). APP itself is heterogeneous, ranging from 110 to 140 kDa, with three major isoforms (695, 751, and 770 residues in length) determined by the splicing pattern of its expression product, and is subjected to a range of post-translational modifications including sulfation, phosphorylation, and N- and O-linked glycosylation (Zheng and Koo, 2011). The generation of amyloid-beta results from a cleavage pathway of APP commonly referred to as the amyloidogenic pathway. This begins with the cleavage of APP by beta-secretase (BACE1), generating two fragments: a C-terminal fragment of APP referred to as C99, and a secreted, soluble N-terminal fragment termed sAPP-β. C99 is subsequently bound by the gamma-secretase complex, comprised of four protein subunits: presenilin (PSEN; PSEN1/PSEN2 isoforms), presenilin enhancer (PEN), Nicastrin, and APH-1. Processing of C99 by gamma-secretase results in a series of sequentially shorter cleavage products, until the amyloid-beta peptide is released (O’Brien and Wong, 2011). In the central nervous system, amyloid-beta is predominantly secreted by neurons and astrocytes into the extracellular space of the brain and physiologically cleared by the vascular system and cerebrospinal fluid. In AD, clearance mechanisms for amyloid-beta are impaired, leading to accumulation in the brain parenchyma (Ramanathan et al., 2015). Multiple amyloid-beta isoforms have been observed in the brain tissue of patients with AD, including amyloid-beta_1–40_ (Aβ40), amyloid-beta_1–42_ (Aβ42), and amyloid-beta_1–43_ (Aβ43; Welander et al., 2009); among them, Aβ42 is the primary constituent of neuritic plaques observed in end-stage AD (O’Brien and Wong, 2011).

**Figure 2:**
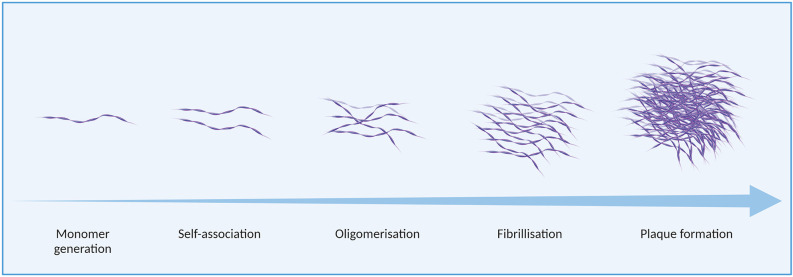
Amyloid-beta aggregation in Alzheimer’s disease (AD).

While physiological roles of amyloid-beta peptides have been identified (Pearson and Peers, 2006; Morley et al., 2019), the aggregation of amyloid-beta monomers has causally been associated with neuronal toxicity (Yankner and Lu, 2009; Pauwels et al., 2012; Prasansuklab and Tencomnao, 2013; Carrillo-Mora et al., 2014). Amyloid-beta aggregates occur in a variety of assemblies, from low molecular weight oligomers (including dimers, trimers, tetramers, and pentamers) to higher molecular weight oligomers (hexamers, nonamers, dodecamers; Wolff et al., 2017), protofibrils, and fibrils, as well as amorphous aggregates (Jiang et al., 2012). *in vitro*, the formation of these aggregate species is affected by various factors including the presence and concentration of specific ions, such as metals, as well as pH and temperature (Valerio et al., 2008; Jiang et al., 2012; Bin et al., 2013; Faller et al., 2013; Bhowmik et al., 2014; Zhao and Ai, 2018). Amyloid-beta aggregation is illustrated in Figure 2.

Multiple regions of the amyloid-beta sequence are considered pro-aggregatory. A report by Liu et al. (2004) described six fragments of amyloid-beta_1–40_ (Aβ40), formed by residues Aβ40_1–28_, Aβ40_12–28_, Aβ40_17–28_, Aβ40_10–20_, Aβ40_25–35_, and Aβ40_17–40_, which had a pro-aggregatory effect on the full-length peptide. Hsu et al. (2018) later identified several key residues involved in amyloid-beta aggregation: H14, E22, D23, G33, G37, and G38. Enache et al. (2018) later characterised the influence of key hydrophobic regions, particularly the well-characterised hydrophobic core domain KLVFF, as well as the C-terminal hydrophobic sequence IIGLMVGGVV and a histidine-containing tetrad, VHHQ, on amyloid-beta aggregation through atomic force microscopy and voltammetry. New insights into the aggregation pathway of amyloid-beta were also attained more recently by Nirmalraj et al. (2020) at nanometer resolution. Aβ42 was shown to aggregate faster than Aβ40 at all stages of assembly. Additionally, the study by Nirmalraj et al. (2020) confirmed that oligomers are not simply an intermediate aggregation species along a linear path to the formation of mature fibrils; oligomers were present even at timepoints where mature fibrils were detected. The pathological effects of different aggregate assemblies have been the subject of extensive research and debate (reviewed by Di Carlo, 2010), however, it is widely accepted that soluble oligomers are a highly cytotoxic species, preceding the development of end-stage neuritic plaques (Resende et al., 2008; Larson and Lesné, 2012; Sengupta et al., 2016; Chen et al., 2017).

### Tau

Tau proteins are encoded by the *MAPT* gene on chromosome 7 (Neve et al., 1986); the pre-mRNA product of *MAPT* undergoes a range of alternative splicing events, resulting in transcript variants encoding six protein isoforms found in the central nervous system (D’Souza and Schellenberg, 2000). These isoforms range from 352 to 441 residues in length and are comprised of four distinct functional domains: the N-terminal projection domain (residues 1–165), proline-rich region (PRR; residues 166–242), microtubule-binding domain (MTBD; 243–367), and C-terminal domain (368–441). The N-terminal domain and MTBD are the primary sites affected by alternative splicing, and the resulting isoforms can be categorised by the number of repeat regions comprising the MTBD: 3R (three-repeat) and 4R (four-repeat) tau (D’Souza and Schellenberg, 2000). The absence of an insert, or the presence of one or two inserts, in the N-terminal projection domain also demarcates each of the isoforms as 0N, 1N or 2N, respectively; the six isoforms are thus referred to as 0N3R, 1N3R, 2N3R, 0N4R, 1N4R, and 2N4R, depending on their number of N-terminal inserts and MTBD repeats. In the adult human brain, 3R and 4R isoforms of tau are present in equal abundance, and tau aggregates found in AD are comprised of both three- and four-repeat isoforms (Kolarova et al., 2012).

**Figure 3:**
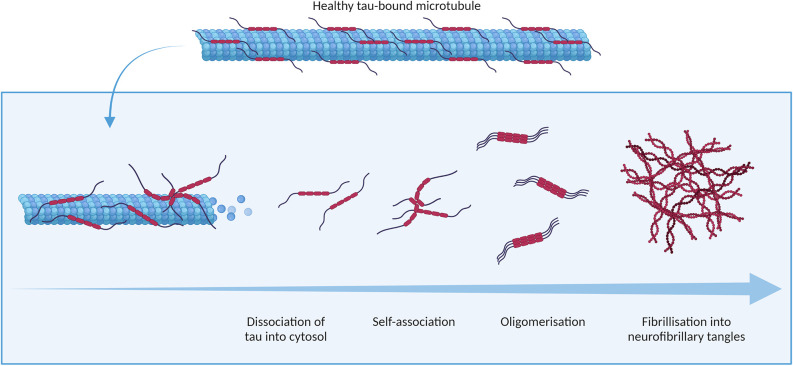
Tauopathy in Alzheimer’s disease.

Physiologically, tau is involved in the assembly and stabilisation of microtubules (Weingarten et al., 1975), as well as in the regulation of intracellular trafficking (Vershinin et al., 2007; Dixit et al., 2008). These activities are regulated by post-translational modifications of the tau protein, which include phosphorylation, glycosylation, deamidation, oxidation, nitration, glycation, and ubiquitination. These modifications to tau and their physiological consequences have previously been reviewed in detail by Avila et al. (2004). Most pertinent to the development of tau aggregates found in AD is tau phosphorylation, which negatively regulates the binding of tau to microtubules and the cellular membrane (Brandt et al., 1995). Tau phosphorylation can occur at any of the multiple serine, threonine and tyrosine residues found on the protein (Williamson et al., 2002; Noble et al., 2013). Phosphorylation of tau induces a conformational change in the protein, reducing its ability to stimulate microtubule assembly (Jameson et al., 1980). In AD, phosphorylation of tau is observed to be over three times higher than in physiological conditions (Köpke et al., 1993). Hyperphosphorylated tau detaches from microtubules, aggregating in the cytosol, as illustrated in Figure 3. Tau acetylation, which similarly increases the negative charge of tau, has also been associated with AD (Irwin et al., 2012; Lucke-Wold et al., 2017).

Tau aggregation primarily involves a shift in the conformation of two hexapeptide regions in the protein, VQIVYK and VQIINK (also referred to as PHF6 and PHF6* respectively), from a random coil to a beta sheet structure (von Bergen et al., 2005; Li and Lee, 2006; Eschmann et al., 2015). Oligomers of tau aggregate further to form paired helical filaments (PHFs) and, subsequently, the neurofibrillary tangles (NFTs) observed in end-stage AD. Tau multimers in a variety of conformations including straight filaments, twisted ribbons, and small oligomeric aggregates have also been observed in the brain tissue of patients with AD (Grundke-Iqbal et al., 1986; Meraz-Ríos et al., 2010). Recent, comprehensive reviews by Niewiadomska et al. (2021) and Shafiei et al. (2017) have established oligomeric assemblies of tau as cytotoxic species.

## Cationic Arginine-Rich Peptides as Aggregation Inhibitors for Alzheimer’s Disease

### Inhibitory Peptides for Amyloid-Beta Aggregation

Through phage display, Kawasaki et al. (2010) identified libraries of three- and four-residue peptides capable of inhibiting (Aβ42) oligomerisation, and found arginine-containing peptides were enriched in both libraries; arginine comprised two of three, and two-to-three of four, residues in the majority of peptides identified, including RRRA, RRRL, RFRK, RRY, and RPR. Kawasaki et al. (2011) also found that while monomeric arginine and di-arginine were able to bind Aβ42, they did not have significant effects on the formation of Aβ42 oligomers, leading the authors to conclude that these molecules were too small to effectively inhibit Aβ42 oligomerisation. Together with the higher binding affinity and inhibitory effect of SRPGLRR in comparison to the three- and four-residue peptides, Kawasaki et al. (2011) concluded the size of the compound could be an important factor in the development of inhibitory peptides for amyloid-beta aggregation.

Notably, among the arginine-rich peptides screened by Kawasaki et al. (2011), RRRA and RRRL were found to be comparatively weaker, yet still effective, inhibitors of Aβ42 oligomerisation than RFRK. While the authors concluded that the higher efficacy of RFRK as an aggregation inhibitor was likely due to the phenylalanine residue of RFRK binding a phenylalanine residue of amyloid-beta, thereby strengthening the interaction between the inhibitor and amyloid-beta, an arginine residue in the second position of either inhibitory peptide could also bind a phenylalanine residue of Aβ42 through its aliphatic side chain, and ability to form cation-pi interactions with aromatic residues. It is, therefore, possible that the increased efficacy of RFRK was rather due to the cationicity of the lysine residue in RFRK imparting a stronger capacity for inhibition of Aβ42 aggregation than the terminal alanine and leucine residues of RRRA and RRRL, respectively. Regardless, the efficacy of RRRA, RRRL, RFRK, and SRPGLRR as inhibitors of Aβ42 oligomerisation led Kawasaki et al. (2011) to consider arginine an important residue for inhibiting Aβ42 aggregation.

Further work by Kawasaki and Kamijo (2012) demonstrated that two additional hybrid peptides combining arginine residues with organic moieties, Arg-Arg-7-amino-4-trifluoromethylcoumarin (RR-AFC) and Arg-Arg-thiobenzyl ester (ZRR-SBzl), were also able to inhibit amyloid-beta aggregation. Recent *in silico* characterisation of RR-AFC conducted by Barale et al. (2019) using molecular dynamics simulation indeed evidenced the critical role of arginine residues in the destabilisation of amyloid-beta protofibrils; in particular, the arginine residues of RR-AFC were found to bind amyloid-beta protofibrils through hydrogen bonding *via* their guanidinium moieties.

Many peptide-based candidates rationally designed to inhibit amyloid-beta aggregation have been derived from the sequence of amyloid-beta itself, exploiting its propensity for self-aggregation to facilitate targeted binding (Watanabe et al., 2002; Austen et al., 2008; Viet et al., 2011; Arai et al., 2014; Kino et al., 2015; Kumar et al., 2015; Cheng et al., 2017; Lu et al., 2019; Jokar et al., 2020). The majority of these peptides contain elements derived from amyloidogenic sequences involved in beta-sheet formation (Moss et al., 2003), such as the KLVFF (amyloid-beta_16–20_) and GGVVIA (amyloid-beta_37–42_) motifs, and are thus termed beta-sheet breakers (Soto et al., 1998). The design of these peptides generally includes an amyloid-derived sequence for target binding, conjugated to a flexible hydrophilic or cationic element to disrupt aggregation. One such inhibitor was a KLVFF-derived retro-inverso peptide, RI-OR2 (rGffvlkGr; lower case denoting D-amino acids), which effectively reduced Aβ42 aggregation *in vitro* (Parthsarathy et al., 2013). As RI-OR2 was not designed to penetrate the blood-brain barrier, it was subsequently fused to the arginine-rich cell-penetrating TAT peptide resulting in the hybrid peptide, RI-OR2-TAT (Ac-rGffvlkGrrrrqrrkkrGy-NH_2_; net charge +11.0). Intriguingly, experimental results from Parthsarathy et al. (2013) indicated that RI-OR2-TAT not only had beneficial effects on the aggregation and cytotoxicity of Aβ42 aggregation in cell models and in a transgenic mouse model of amyloidogenesis (APPSwe/PS1ΔE9) *in vivo*, but also demonstrated greater efficacy for inhibiting the aggregation of Aβ42 *in vitro* compared to RI-OR2. The inhibitory effects of RI-OR2-TAT on Aβ42 aggregation were evident at even the earliest detectable stages of Aβ42 oligomerisation. Surface plasmon resonance (SPR) experiments conducted by Parthsarathy et al. (2013) further indicated RI-OR2-TAT had a higher binding affinity for Aβ42 than RI-OR2 alone. It is likely that the increased efficacy of RI-OR2-TAT as an inhibitor of Aβ42 aggregation was mediated by the arginine content of TAT, specifically through: (a) greater capacity for hydrogen bonding imparted by the arginine residues of the TAT peptide, facilitating a higher number of electrostatic interactions between the inhibitory peptide and Aβ42; (b) increased cationic charge of the RI-OR2-TAT peptide conferred by the arginine-rich TAT component, driving further charge-based repulsion of bound Aβ42 monomers; and (c) the increased length of the inhibitory peptide likely causing greater steric and/or electrostatic interference between monomers of Aβ42, preventing their self-association. Importantly, these findings suggested that arginine-rich peptides could themselves have a mechanistic role in modulating the development of amyloid-beta aggregates beyond their use as “carrier” peptides alone.

Cheng et al. (2017) later developed a bipartite peptide comprised of polyarginine-8 (R8) conjugated to the sequence of amyloid-beta_25–35_ (RRRRRRRRGSNKGAIIGLM; net charge +9.0) which effectively reduced cerebral accumulation of amyloid-beta and ameliorated cognitive deficits in the *APP/PS1* double transgenic mouse model of AD. The design of the peptide was comprised of the amyloid-beta_25–35_ self-recognition element to facilitate binding to amyloid-beta monomers, with the polyarginine component intended to drive charge-based repulsion of the bound monomers from each other, preventing their ability to aggregate.

More recently, Henning-Knechtel et al. (2020) designed two cell-penetrating peptides comprised of a hydrophobic signal sequence (MLRTKDLIWTLFFLGTAVS; NCAM1) conjugated to a polycationic sequence (KKRPKP; PrP_23–28_) or an amyloid-derived self-recognition motif sequence (KLVFF; Aβ_16–20_) *via* a lysine residue to increase the cationic charge of the peptide. The NCAM1-PrP peptide carried a higher overall charge of +6 compared to the +4 charge of NCAM1-Aβ, resulting from a higher proportion of cationic residues. While both NCAM1-PrP and NCAM1-Aβ were found to inhibit the aggregation of Aβ42 *in vitro*, NCAM1-PrP achieved effective inhibition at substoichiometric concentrations, while an equimolar concentration of NCAM1-Aβ to amyloid-beta was required. As amyloid-beta is negatively charged at physiological pH (net charge −2.7), the increased efficacy of NCAM1-PrP as an aggregation inhibitor compared to the KLVFF-containing NCAM1-Aβ peptide was attributed to its higher proportion of cationic residues, reportedly essential in stabilising the dimers formed between NCAM1-PrP and amyloid-beta. Additionally, although NCAM1 itself was able to bind amyloid-beta, the addition of a polycationic sequence was required to exert a modulatory effect on aggregation, suggesting the overall charge of an inhibitory peptide may be as important for preventing the aggregation of amyloid-beta as structural specificity. Indeed, Henning-Knechtel et al. suggested that the NCAM1-Aβ peptide could be optimised through further addition of cationic residues, thus it is conceivable that higher arginine content could increase its efficacy. These results also support the idea that electrostatic interactions between an inhibitory peptide and amyloid-beta are sufficient to drive target binding in the absence of amyloid-beta-derived sequence motifs, as was demonstrated by Parthsarathy et al. (2013).

The effects of a short peptide comprised entirely of arginine, polyarginine-9 (R9), were investigated by Fonar et al. (2018) in the triple transgenic (3xTg) mouse model of AD which harbours FAD mutations inducing the development of both amyloid-beta and tau pathology. 3xTg mice treated with R9 displayed a trend toward lower levels of cerebral amyloid-beta compared to untreated controls, as well as a trend toward reduced levels of oligomeric amyloid-beta in hippocampal lysates, however, these findings did not reach statistical significance. The effects of longer polyarginine peptides on amyloid-beta pathology may be an interesting area for further research; it is possible that increased cationic charge imparted by longer stretches of arginine could impart a greater capacity for electrostatic repulsion of amyloid-beta monomers, thereby inhibiting aggregation. Additionally, D-enantiomeric polyarginine peptides could potentially exert a stronger effect due to the increased proteolytic stability of D-amino acids (Feng and Xu, 2016) in comparison to L-isoforms.

Among the most promising aggregation inhibitors targeting amyloid-beta is the cationic arginine-rich RD2 peptide (ptlhthnrrrrr; +6.2 net charge), demonstrated to eliminate amyloid-beta oligomers *in vivo*, and rescue amyloid-beta pathology and cognitive deficits in a transgenic mouse model of AD (Schemmert et al., 2019; Zhang et al., 2019). RD2 is a rationally-optimised derivative of an arginine-rich precursor peptide, D3 (rprtrlhhrnr; +5.2 net charge), which was initially identified through mirror- image phage display (Wiesehan et al., 2003) and also shown to be effective in targeting amyloid-beta (van Groen et al., 2008). A series of C-terminally amidated D3 analogues were developed, outlined in Table 1, to identify a candidate for further development (Klein et al., 2017). Notably, increasing the charge of the inhibitory peptide was associated with increased binding affinity to amyloid-beta (Ziehm et al., 2016).

**Table 1:** Cationic arginine-rich peptides with modulatory effects on amyloid-beta aggregation.

Peptide	Sequence (Net charge at pH 7)	Effect	Reference
R5	RRRRR (+5.0)	Modulation of Aβ aggregation towards the formation of large, amorphous, non-toxic aggregates	Gibson and Murphy (2005)
KLVFF-R5	KLVFFRRRRR (+6.0)
D3	rprtrlhthrnr (+5.2)	Reduction of Aβ oligomers *in vitro*	van Groen et al. (2008)
RFRK	RFRK (+3.0)	Inhibition of Aβ aggregation *in vitro*	Kawasaki et al. (2011)
RRRL	RRRL (+3.0)
RRRA	RRRA (+3.0)
SRPGLRR	SRPGLRR (+3.0)
RR-AFC	RR-7-amino-4-trifluoromethylcoumarin (+3.0)	Inhibition of Aβ aggregation *in vitro*	Kawasaki and Kamijo (2012)
ZRR-SBzl	RR-thiobenzyl ester (+3.0)
RI-OR2-TAT	Ac-rGffvlkGrrrrqrrkkrGy-NH_2_ (+11.0)	Inhibition of Aβ aggregation *in vitro*,reduced cerebral Aβ load *in vivo*	Parthsarathy et al. (2013)
15M S.A.	Ac-rklmqptrnrrnpnt-NH_2_ (+5.0)	Modulation of Aβ aggregation towards the formation of large, amorphous, non-toxic aggregates	Barr et al. (2016)
DB1	rpitrlhtdrnr-NH_2_ (+4.1)	Inhibition of Aβ aggregation *in vitro*	Klein et al. (2017)
DB2	rpittlqthqnr-NH_2_ (+3.1)
DB3	rpitrlrthqnr-NH_2_ (+5.1)
DB4	rprtrlrthqnr-NH_2_ (+6.1)	No effect on Aβ aggregation *in vitro*
DB5	rpitrlqtheqr-NH_2_ (+3.1)	Inhibition of Aβ aggregation *in vitro*
DB3DB3	rpitrlrthqnrrpitrlrthqnr-NH_2_ (+9.2)
RD2RD2	ptlhthnrrrrrptlhthnrrrrr (+11.4)	Reduction of Aβ oligomers *in vitro* and *in vivo*	Kutzsche et al. (2017)
RD2D3	ptlhthnrrrrrrprtrlhthrnr-NH_2_ (+11.4)
D3D3	rprtrlhthrnrrprtrlhthrnr-NH_2_ (+11.4)
R8-Aβ_25_–_35_	RRRRRRRR-GSNKGAIIGLM (+9.0)	Reduced cerebral Aβ load *in vivo*	Cheng et al. (2017)
R9	RRRRRRRRR (+9.0)	Trend toward reduced Aβ oligomers *in vivo*	Fonar et al. (2018)
RD2	ptlhthnrrrrr-NH_2_ (+6.2)	Reduction of Aβ oligomers *in vitro* and *in vivo*	Zhang et al. (2019)
NCAM1-PrP	MLRTKDLIWTLFFLGTAVS-KKRPKP-NH_2_ (+6.0)	Inhibition of Aβ aggregation *in vitro*	Henning-Knechtel et al. (2020)
NCAM1-Aβ	MLRTKDLIWTLFFLGTAVS-KKLVFF-NH_2_ (+4.0)		

*Lowercase letters denote D-enantiomeric residues*.

Continued characterisation of D3 analogues indicated RD2 had the most favourable pharmacokinetic profile with regards to half-life and oral bioavailability (Leithold et al., 2016). Indeed, the initial success of RD2 in phase I clinical trials has led to its ongoing development as a potential therapeutic candidate for AD (Elfgen et al., 2021). The molecular mechanisms of both D3 and RD2 were investigated in detail through computational studies and experimental verification by Olubiyi et al. (2014), revealing that both D3 and RD2 bind amyloid-beta with high affinity, and reduce β-sheet formation, largely due to electrostatic interactions between the arginine residues of the inhibitory peptides and key anionic residues (E11, E22 and D23) of amyloid-beta.

### Inhibitory Peptides for Tau Aggregation

Peptide-based inhibitors of tau aggregation have focused on the PHF6 and PHF6* regions of the MTBD, which are well-characterised as pro-aggregatory sequences (Li and Lee, 2006; Eschmann et al., 2015). Ralhan et al. (2017) reported inhibition of PHF6 aggregation by the poly-arginine-6 (R6) and R8 peptides, as well as the reversal of PHF6 fibril formation by R6 which acted as a beta-sheet breaker, inducing the disassembly of PH6 fibrils into sparser aggregates. While early peptides targeting the PHF6 region have shown effective inhibition of the isolated hexapeptide *in vitro*, these peptides were unable to prevent the aggregation of full-length tau (Sievers et al., 2011; Zheng et al., 2011; Seidler et al., 2018), which additionally contains the amyloidogenic PHF6* sequence considered to be a significant driver of tau aggregation. Seidler et al. (2018) subsequently developed new inhibitory peptides through combinatorial mutagenesis of the VQIINKKLD motif. These peptides are comprised of 10 residues, outlined in Table 2, and able to prevent aggregation of the full-length tau protein. Importantly, to achieve this, the inclusion of an arginine residue was required at the ninth position in order to prevent self-association of one of the two main interfaces involved in full-length tau aggregation: the KKL region of the tau KVQIINKKLD sequence, termed “interface B”.

**Table 2:** Polyarginine and arginine-rich peptides with modulatory effects on tau aggregation.

Peptide	Sequence (Net charge at pH 7)	Effect	Reference
R6	RRRRRR (+6.0)	Inhibition of tau PHF6 aggregation *in vitro*	Ralhan et al. (2017)
R8	RRRRRRRR (+9.0)
R32	R_32_ (+32.0)	Inhibition of tau PHF6 and PHF6* aggregation *in vitro*	Nadimidla et al. (2017)
R96	R_96_ (+96.0)
WINK	DVQWINKKRK (+3.0)	Inhibition of full-length tau aggregation *in vitro*	Seidler et al. (2018)
MINK	DVQMINKKRK (+3.0)
AG01	Ac-RGVQIINKGR-NH_2_ (+3.0)	Reduction of tau Δ1–250 aggregation *in vitro*	Aggidis (2019)
AG02	Ac-RGVQIVYKGR-NH_2_ (+3.0)		
AG02R4	Ac-RRGVQIVYKGRR-NH_2_ (+5.0)		
AG02R5	Ac-RGVQIVYKGRRRR-NH_2_ (+6.0)		
AGR502	Ac-RRRRGVQIVYKGR-NH_2_ (+6.0)		
AG02PR5	Ac-RRRGVQIVYKGRRRR-NH_2_ (+8.0)		
AG02R6	Ac-RRRGVQIVYKGRRR-NH_2_ (+7.0)		
AG02R9	Ac-RGVQIVYKGRRRRRRRR-NH_2_ (+10.0)		
AG02TAT	Ac-RGVQIVYKGRYGRKKRRQRRR-NH_2_ (+11.0)		
AG02ΔI	Ac-RGVQK(Ac)VYKGR-NH_2_ (+3.0)		
AG02ΔV	Ac-RGVQIK(Ac)YKGR-NH_2_ (+3.0)		
AG03	Ac-RGVQIK(Ac)YKPGRRRRRRRR-NH_2_ (+10.0)		
AG03-Cys	Ac-RGVQIK(Ac)YKPGRRRRRRRRC-OH (+9.9)		
AG03M	Ac-RGV(m)QI(m)K(Ac)Y(m)KP(m)GRRRRRRRR-NH_2_ (+10.0)		
FAM-RI-AG03	Ac-k(FAM)rrrrrrrrGpkyk(ac)iqvGr-NH_2_ (+10.0)		
Scrambled AG03	Ac-RGQPKIIK(Ac)YVGRRRRRRRR-NH_2_ (+10.0)	No significant effect on tau Δ1–250 aggregation	
R8	RRRRRRRR (+8.0)		
TAT	Ac-YGRKKRRQRRR-NH_2_ (+8.0)	
p-NH	NITMNSRRRRNH (+4.1)	Reduction of tau PHF6 aggregation *in vitro* and tau aggregation *in vivo*	Zhang et al. (2020)
RI-AG03	Ac-rrrrrrrrGpkyk(ac)iqvGr-NH_2_ (+10.0)	Inhibition of tau PHF6 and tau Δ1–250 aggregation *in vitro*	Aggidis et al. (2021)
TAT-7H	YGRKKRRQRRR-HHHHHHH (+8.7)	Inhibition of tau Ser202 and Thr205 phosphorylation	Kondo et al. (2021)

*(Ac) and (m) denote residue acetylation and methylation, respectively. Lowercase letters denote D-enantiomeric residues*.

Longer polyarginine peptides (R32 to R96) were demonstrated by Nadimidla et al. (2017) to inhibit the aggregation of both PHF6 and the PHF6*-containing amyloidogenic tau fragment GKVQIINKLDL, as well as aggregation of the full-length mutant tau protein P301L, which is found in human tauopathies (Cosacak et al., 2017). The aggregation of PHF6 and PHF6* peptides was also inhibited, albeit to a lesser degree, by the cationic polymer polyethylenimine (PEI), implicating the positive charge of these compounds as a key factor in suppressing tau aggregation. Tau is aggregated in the presence of heparin *in vitro* to model the proaggregatory role of polyanionic molecules such as heparan sulfate proteoglycans found in NFTs *in vivo* (Fichou et al., 2018; Maïza et al., 2018); it is likely that, in addition to interacting electrostatically with negatively charged stretches of tau itself, strongly cationic compounds such as polyarginine could inhibit the seeding and nucleation of tau aggregation by complexing with essential polyanionic cofactors involved in the aggregation process. Furthermore, post-translational modifications which increase the negative charge of tau *in vivo*, such as phosphorylation, acetylation and nitration, could potentially increase the affinity of CARPs for tau, and their ability to modulate its aggregation.

Zhang et al. (2020) subsequently reported an arginine-rich, D-enantiomeric peptide termed p-NH (nitmnsrrrrnh; net charge +4.1), discovered through phage display, was able to inhibit tau aggregation *in vitro* and reduce tau^P301S^ levels in transgenic mice. *In vitro*, p-NH was shown to inhibit PHF6 aggregation in a dose-dependent manner; this effect was optimal at an 8-fold molar excess of the peptide over PHF6, but remained effective at equimolar and substoichiometric concentrations. Remarkably, p-NH was also able to reverse aggregation when added to preformed PHF6 fibrils. Importantly, Zhang et al. (2020) reported p-NH was able to interact directly with PHF6 through hydrogen bond formation.

More recently, Aggidis et al. (2021) reported a D-enantiomeric, retro-inverso peptide (rrrrrrrrGpkyk(ac)iqvGr; net charge +11.0) based on the PHF6 sequence, termed RI-AG03, able to prevent tau aggregation. As a retro-inverso peptide, RI-AG03 was optimised for increased proteolytic stability from the AG03 peptide, itself selected from a family of peptides designed to inhibit tau aggregation (Aggidis, 2019) which are summarised in Table 2. The majority of the inhibitory peptides developed by Aggidis (2019) significantly reduced the aggregation of recombinant human tau Δ1–250, which exhibits faster aggregation kinetics than full-length tau protein, in a dose-dependent manner. Importantly, Aggidis (2019) reported that increasing arginine content of the inhibitory peptides, up to five residues, increased tau Δ1–250 solubility. An interesting observation is that R8 alone was unable to prevent tau Δ1–250 aggregation, in contrast to prior results indicating the effective inhibition of PHF6 aggregation by R6 and R8 (Ralhan et al., 2017); taken together, these effects are consistent with observations reported by Seidler et al. (2018) that PHF6* aggregation is a significant driver of aggregation in larger tau fragments.

## Cationic Arginine-Rich Peptides as Modulators of Proteopathic Cytotoxicity

As the precise, mechanistic roles of amyloid-beta and tau aggregates in the pathogenesis of AD remain unclear, CARPs could also be beneficial by mitigating the cellular effects of cytotoxic amyloids, rather than preventing the formation of aggregates *per se*. As comprehensively detailed in a review by Meloni et al. (2020), CARPs have favourable biological effects in models of neuronal injury and disease, such as stroke and traumatic brain injury, through multimodal mechanisms of action. Here, we will discuss how the bioactive properties of CARPs could be beneficial specifically in the context of proteopathic cytotoxicity associated with AD.

### Modulation of Cytotoxicity Through Effects on Amyloid Formation

There are multiple potential mechanisms for CARPs to confer cytoprotection against toxic aggregates of amyloid-beta and tau. As described, the predominant approach has focused on inhibiting aggregation; CARPs which have demonstrated the ability to reduce amyloid-beta-induced cytotoxicity *in vitro* by inhibiting aggregation include those developed by Kawasaki et al. (2011), Parthsarathy et al. (2013), Cheng et al. (2017) and Henning-Knechtel et al. (2020). Additionally, Nadimidla et al. (2017), whose work reported inhibition of tau aggregation through high molecular-weight polyarginine peptides, demonstrated increased rates of cell survival in cultures exposed to cytotoxic concentrations of tau when treated with polyarginine peptides.

However, as the cytotoxicity of amyloid-beta aggregates varies depending on the aggregate conformation, inhibiting aggregation is not the only means of decreasing the concentration of cytotoxic aggregate species. CARPs, through their ability to electrostatically bind monomers of amyloid-beta, may be able to bind and stabilise monomers in conformations favouring non-oligomeric aggregation pathways. Gibson and Murphy (2005) previously reported that polyarginine-5 (R5) and KLVFF-R5 peptides broadly increased the aggregation of amyloid-beta_1–42_ overall, resulting in the formation of larger amyloid-beta_1–42_ aggregates with lower cytotoxicity. Barr et al. (2016) reported that a 15-residue cationic peptide, with 20% of its sequence comprised of arginine residues, reduced the formation of soluble, cytotoxic amyloid-beta_1–42_ oligomers by driving the aggregation pathway toward the formation of larger, amorphous aggregates; these amorphous aggregates were also observed to have lower cytotoxicity than the oligomers formed by amyloid-beta_1–42_ alone. A potential therapeutic mechanism for peptides targeting amyloid-beta could therefore involve the reduction of cytotoxic, soluble oligomeric amyloid-beta species by driving the aggregation pathway toward the formation of non-toxic insoluble aggregate species, rather than aiming to inhibit aggregation.

### Potential Indirect Mechanisms of Cytoprotection Against Amyloids

CARPs may be able to prevent the cellular conditions favouring the formation of cytotoxic aggregates. For example, a proteomic study of neuronal cultures treated with the polyarginine-18 peptide (R18) showed overall levels of tau were significantly decreased (MacDougall et al., 2019a, supplementary information), however the mechanisms underlying this effect are unclear. Arginine is also able to scavenge free radicals and mitigate oxidative stress (Wascher et al., 1997; Haklar et al., 1998), an ability that extends to polyarginine peptides (Marshall et al., 2015). Oxidative stress has a synergistic relationship with amyloid-beta pathology (reviewed in detail by Cheignon et al., 2018), as well as tau phosphorylation and polymerisation (reviewed by Zhao and Zhao, 2013). Reducing oxidative stress could potentially therefore aid, indirectly, in preventing the formation of aggregate species. Additionally, as oxidative stress is believed to be one of the main mechanisms through which amyloid-beta and tau aggregates induce toxic effects (Butterfield et al., 2013), there may be a potential role for arginine and CARPs in cytoprotection through the indirect, downstream effects of rescuing oxidative damage, an area which requires further investigation.

CARPs may also be able to prevent the development of tauopathy through inhibition of tau hyperphosphorylation, which is pro-aggregatory. The p-NH peptide developed by Zhang et al. (2020) was reported to significantly reduce tau phosphorylation at Thr181, Ser202, Thr231, Ser396, and Ser404 in the human neuroblastoma N2a cell line. More recently, a CARP developed by Kondo et al. (2021) termed TAT-7H (YGRKKRRQRRR-HHHHHHH; net charge +8.7) was shown to inhibit the phosphorylation of Ser202 and Thr205 in a neuronal cell line differentiated from human tau^P301S^ double knock-in induced pluripotent stem cells.

**Figure 4:**
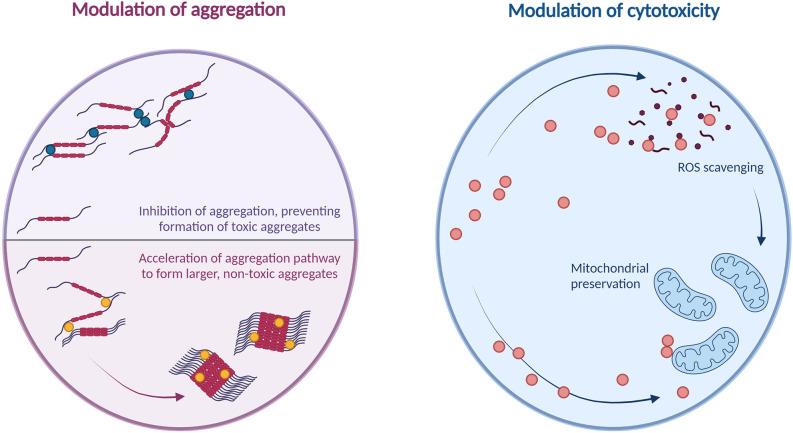
Summary of potential cytoprotective mechanisms of arginine and cationic arginine-rich peptides against amyloids.

Protein aggregates in AD also induce cytotoxicity through mitochondrial damage; tau oligomers are believed to induce mitochondrial damage *via* their detrimental effects on intracellular transport networks (Shafiei et al., 2017), while accumulation of amyloid-beta at the mitochondrial membranes induces mitochondrial damage through mechanisms including aberrant interactions with mitochondrial proteins, generation of reactive oxygen species, and disruption of the electron transport chain (reviewed in detail by Reddy et al., 2010, and Chen and Yan, 2007). The ability of CARPs to preserve mitochondrial function has been discussed at length in a review by MacDougall et al. (2019b); thus, another area for future research would be the potential for CARPs to preserve mitochondrial function and mitigate cytotoxicity in models of amyloid-beta and tau-induced neuronal injury. These mechanisms are summarized in Figure 4.

It is also possible that arginine-rich peptides, through their extensive capacity for electrostatic interactions, may be able to bind pre-formed aggregates and prevent their deleterious cellular effects. It was recently discovered that fibrillar formations of tau exhibit an altered interactome than tau monomers and nano-aggregates. Ferrari et al. (2020) demonstrated that tau fibrils preferentially interact with a set of proteins containing disordered stretches significantly enriched for arginine residues, with these aberrant interactions mediated by pi-pi stacking interactions. Crucially, Ferrari et al. (2020) found that the replacement of arginine residues in these interacting proteins with lysine precluded their interactions with tau fibrils. The proteins comprising the altered interactome of tau fibrils were identified as predominantly belonging to three functional clusters: RNA-binding proteins, regulators of protein phosphorylation, and microtubule-associated proteins. It was inferred that these interactions may, in part, be responsible for the cytotoxicity of tau aggregates in AD (Ferrari et al., 2020). It is possible that exogenous arginine-rich compounds may be able to prevent cytotoxicity by competitively binding to tau fibrils through the same mechanism, preventing tau fibrils from associating with these aberrant interactors. Thus, the potential effects of CARPs on the cytotoxicity of preformed tau aggregates presents an interesting area for future research.

## Potential Roles for Arginine in Other Neurodegenerative Disease-Associated Proteopathies

Monomeric arginine has demonstrated efficacy in preventing the aggregation of alpha-synuclein. While alpha-synuclein aggregation is predominantly associated with Parkinson’s disease (reviewed comprehensively by Fields et al., 2019), alpha-synuclein has also been implicated in AD (Kotzbauer et al., 2001; Crews et al., 2009; Twohig and Nielsen, 2019). Arginine was found to inhibit rotenone-induced aggregation of alpha-synuclein *in vitro*, even inhibiting further aggregation even when added past the stage of initial nucleation (Shristi, 2014). The inhibitory effects of arginine on alpha-synuclein aggregation were further confirmed in detail by Ghosh et al. (2018), who demonstrated arginine-based inhibition of alpha-synuclein aggregation both *in vitro* and *in situ* in live cells. Ghosh et al. (2018) also evidenced the cytoprotective effects of arginine on cultures treated with pre-aggregated alpha-synuclein. Interestingly, the use of lysine, a similarly cationic amino acid, was associated with increased alpha-synuclein aggregation *in vitro*, while guanidinium hydrochloride was associated with decreased viability of cells treated with aggregated alpha-synuclein. These results indicate that while the guanidinium moiety of arginine is essential to its activities, the overall properties of arginine rather than either its general cationicity or the guanidinium moiety in isolation, are key to its anti-aggregatory and cytoprotective effects in the context of alpha-synuclein aggregation.

Recently, the CARP RD2RD2 (ptlhthnrrrrrptlhthnrrrrr; +10.4 charge) was investigated as a potential therapeutic candidate for amyotrophic lateral sclerosis (ALS) in a mouse model of mutant superoxide dismutase 1 (SOD1) expression (Post et al., 2021). Administration of RD2RD2 was associated with reductions in motor deficits and neuroinflammation, although the precise molecular mechanisms underlying these effects remain to be elucidated.

Minakawa and Nagai (2021) recently discussed arginine as a modulator of aggregatory proteins containing polyglutamine (polyQ) stretches, a pathogenic feature of neurodegenerative diseases such as Huntington’s disease (HD) and spinocerebellar ataxias (SCAs). Oligomers of polyQ proteins, as well as polyQ protein monomers which are enriched for beta-sheet structure, have previously been associated with cytotoxicity (Kayed et al., 2003; Miller et al., 2011). When added to polyQ proteins *in vitro*, monomeric arginine was found to prevent the aggregation of polyQ proteins, including the initial transition from alpha-helical to beta-sheet-enriched monomers, subsequent oligomerisation, and the seeding of aggregation by pre-formed aggregates (Minakawa et al., 2020). Arginine, arginine methyl ester and arginine ethyl ester were also shown by Singh et al. (2019) to prevent aggregation of a polyQ-containing Huntington exon 1 protein (mHTTex1) *in vitro*, and rescue motor deficits in a model of HD *in vivo*. Minakawa et al. (2020) also investigated the effects of arginine administration *in vivo* and found motor deficits were rescued in multiple mouse and invertebrate models of SCA. Notably, arginine was able to rescue motor deficits even past the stage of initial symptom onset in a SCA1 mouse model (Minakawa et al., 2020).

Aggregation of an mHTT protein was also suppressed by a hybrid peptide, 8R10Q (RRRRRRRRQQQQQQQQQQ; net charge +8.0) designed by He et al. (2019) through a principle similar to the bipartite peptide designed for amyloid-beta aggregation by Cheng et al. (2017): inclusion of a self-recognition component (a ten-residue stretch of glutamine), conjugated to a polyarginine component to drive charge-based repulsion of bound monomers as well as increase solubility and membrane permeability.

The effects of arginine and its derivatives on polyQ aggregation and cytotoxicity are consistent with the advantages conferred by the structure and cationicity of arginine in amyloid-beta and tau-targeting peptides, as well as the effects of CARPs including polyarginine peptides on glutamic acid-induced excitotoxicity (reviewed comprehensively by Meloni et al., 2020). By evaluating a set of arginine derivatives with either N- or C- terminal substitutions, Singh et al. (2019) inferred the guanidino group of arginine was essential to its anti-aggregatory effect on a polyQ peptide (polyglutamine-35; Q35) *in vitro*. Citrulline, ornithine and lysine, which lack the guanidino moiety, were unable to prevent Q35 aggregation, while arginine and arginine ethyl ester prevented Q35 aggregation in a dose-dependent manner (Singh et al., 2019).

## Peptidylarginine Citrullination and Arginine-Rich Therapeutics

Arginine molecules can be converted to citrulline by arginine deiminase (ADI) through hydrolysis of their guanidinium groups, causing the strongly basic, cationic arginine side chain to be replaced by neutral urea. Whereas ADI converts free arginine molecules, peptidylarginine deiminases (PADs) catalyse the conversion of peptide-bound arginine residues to citrulline residues (Wang and Wang, 2013). Citrullination affects the ability of the residue to form hydrogen bonds, altering its intermolecular interactions.

Increased PAD activity has been associated with AD (Acharya et al., 2012; Wang et al., 2021); in particular, levels of PAD II detected in the hippocampus were found to be significantly higher in AD patients than in controls by Ishigami et al. (2005), concomitant with the presence of citrullinated proteins in AD-affected hippocampi. It is conceivable that higher levels of PAD activity in AD could affect the overall efficacy of CARP therapeutics through citrullination of their arginine residues, however, this remains to be observed. Moreover, understanding of PADs in AD more broadly remains limited. Further research on peptidylarginine citrullination in AD is required to hypothesise the potential effects of PADs on CARPs as therapeutic candidates in this context. Additionally, as current preclinical models of AD are unable to recapitulate all aspects of AD pathogenesis, it is difficult to predict how overexpression of PADs upregulated in human AD might affect CARPs shown to be highly efficacious in animal models, such as RD2.

It is worth noting, however, that PAD activity is tightly controlled by calcium ions (Lamensa and Moscarello, 1993; Sambandam et al., 2004; Mondal and Thompson, 2019), and PAD II is activated through elevated intracellular calcium levels (Slade et al., 2015). CARPs themselves have previously been shown to reduce intracellular calcium influx through multiple mechanisms (Meloni et al., 2015a,b; Edwards et al., 2017), including downregulation of the NMDAR subunit NR2B. Therefore, it is possible that CARPs may be able to prevent excessive activation of PADs in AD through upstream inhibition of calcium influx. In summary, although PADs could theoretically affect the ability of CARPs to exert beneficial effects in AD through citrullination of their arginine residues, it is difficult to predict whether this is likely to occur *in vivo* based on the paucity of evidence; additionally, the putative effects of CARPs on calcium signalling in states of neurological injury and disease render this a complex area. Regardless, the favourable safety profile of CARPs (Edwards et al., 2020) should allow CARPs to be evaluated at varying dose levels for efficacy in human AD.

## Conclusion

Arginine has several distinctive properties, largely owing to the unique structure and chemistry of the guanidinium group. While monomeric arginine has long been regarded as a potent inhibitor of protein aggregation, polypeptides enriched for arginine residues also display interesting effects on the formation and cytotoxicity of protein aggregates. Arginine residues have substantial bioactive properties in the context of modulating protein aggregation, and should therefore be given particular consideration in the rational design of amyloid-targeting therapeutic peptides.

The lack of effective therapeutic options for AD presents a significant global health challenge. Peptide drugs are a rapidly growing class of therapeutic candidates, and while their design and optimisation carries a distinct set of challenges, they hold promise in the treatment of AD through their potential advantages of high target specificity and bioactivity. We have identified a number of CARPs which effectively modulate the aggregation pathway of amyloid-beta, as well as potential roles of arginine in peptide-based therapeutic design for targeting tau oligomerisation.

Interestingly, CARPs have also demonstrated significant neuroprotective effects in models of stroke and traumatic brain injury, particularly by targeting excitotoxicity, which is also a pathological feature of AD (reviewed by Wang and Reddy, 2017). The complex and multifactorial nature of AD pathogenesis particularly warrants therapeutic candidates with multiple mechanisms of action. The potential for CARPs to modulate proteopathies associated with AD, therefore, warrant further investigation. CARPs could potentially target the formation and/or deleterious cellular effects of amyloids in AD through diverse roles: decreasing the concentration of soluble, cytotoxic oligomers by either preventing aggregation or driving aggregation toward the formation of non-cytotoxic species; mitigating oxidative stress, which is known to drive the formation of cytotoxic amyloids; and preserving the function of mitochondria, which are a site of cellular damage from amyloids. These possibilities present several lines of inquiry for further research.

## Author Contributions

SM wrote the manuscript with input from BM. Both authors revised the manuscript and approved the final version.

## Conflict of Interest

BM is a named inventor of several patent applications regarding the use of CARPs as neuroprotective agents, and a shareholder in Argenica Therapeutics, a company developing R18 as a neurotherapeutic. The remaining author declares the absence of any commercial or financial relationships that could be construed as a potential conflict of interest.

## Publisher’s Note

All claims expressed in this article are solely those of the authors and do not necessarily represent those of their affiliated organizations, or those of the publisher, the editors and the reviewers. Any product that may be evaluated in this article, or claim that may be made by its manufacturer, is not guaranteed or endorsed by the publisher.
